# Toxicity of Nine (Doped) Rare Earth Metal Oxides and Respective Individual Metals to Aquatic Microorganisms *Vibrio fischeri* and *Tetrahymena thermophila*

**DOI:** 10.3390/ma10070754

**Published:** 2017-07-05

**Authors:** Imbi Kurvet, Katre Juganson, Heiki Vija, Mariliis Sihtmäe, Irina Blinova, Guttorm Syvertsen-Wiig, Anne Kahru

**Affiliations:** 1 Laboratory of Environmental Toxicology, National Institute of Chemical Physics and Biophysics, Akadeemia tee 23, 12618 Tallinn, Estonia; imbi.kurvet@kbfi.ee (I.K.); katre.juganson@kbfi.ee (K.J.); heiki.vija@kbfi.ee (H.V.); mariliis.sihtmae@kbfi.ee (M.S.); irina.blinova@kbfi.ee (I.B.); 2 School of Science, Tallinn University of Technology, Ehitajate tee 5, 19086 Tallinn, Estonia; 3 Ceramic Powder Technology AS, Kvenildmyra 6, 7093 Tiller, Norway; guttorm.syvertsen@cerpotech.com; 4 Estonian Academy of Sciences, Kohtu 6, 10130 Tallinn, Estonia

**Keywords:** lanthanides, nanoparticles, doped metal oxides, speciation, hazard evaluation, bioluminescence, cellular membrane integrity, viability, ciliates, Microtox™

## Abstract

Despite the increasing use of rare earth elements (REEs) and oxides (REOs) in various technologies, the information on their ecotoxicological hazard is scarce. Here, the effects of La^3+^, Ce^3+^, Pr^3+^, Nd^3+^, Gd^3+^, CeO_2_, and eight doped REOs to marine bacteria *Vibrio fischeri* and freshwater protozoa *Tetrahymena thermophila* were studied in parallel with REO dopant metals (Co^2+^, Fe^3+^, Mn^2+^, Ni^2+^, Sr^2+^). The highest concentrations of REOs tested were 100 mg/L with protozoa in deionized water and 500 mg/L with bacteria in 2% NaCl. Although (i) most REOs produced reactive oxygen species; (ii) all studied soluble REEs were toxic to bacteria (half-effective concentration, EC_50_ 3.5–21 mg metal/L; minimal bactericidal concentration, MBC 6.3–63 mg/L) and to protozoa (EC_50_ 28–42 mg/L); and (iii) also some dopant metals (Ni^2+^, Fe^3+^) proved toxic (EC_50_ ≤ 3 mg/L), no toxicity of REOs to protozoa (EC_50_ > 100 mg/L) and bacteria (EC_50_ > 500 mg/L; MBC > 500 mg/L) was observed except for La_2_NiO_4_ (MBC 25 mg/L). According to kinetics of *V. fischeri* bioluminescence, the toxicity of REEs was triggered by disturbing cellular membrane integrity. Fortunately, as REEs and REOs are currently produced in moderate amounts and form in the environment insoluble salts and/or oxides, they apparently present no harm to aquatic bacteria and protozoa.

## 1. Introduction

Chemically uniform group of metals, lanthanides (La–Lu), form together with yttrium (Y) and scandium (Sc) the group of REEs. REEs, in particular REOs, are increasingly used in many fields, e.g., catalysis, electronics, wind power generators, glass polishing and ceramics, metallurgical additives and alloys, high strength magnets, fuel cells, gas separation membranes, and fuel additives [1,2,3]. Probably the most rapid increase of REEs production is expected for neodymium (Nd) and dysprosium (Dy), as they are used in magnets of wind turbines and electric/hybrid cars [4,5]. In addition to use in industry, there is a long-term practice of the application of REEs-based micro-fertilizers, and REEs may also be introduced to environment during the production and application of phosphorous fertilizers [6,7,8,9]. Some REEs, such as gadolinium (Gd), are also used in health-related applications: in particular, Gd chelates are used as contrast agents for magnetic resonance imaging [10] and may reach the environment via waste streams. Indeed, increase of REEs concentrations (especially gadolinium) in water bodies due to the anthropogenic activities over the past two decades has been reported [11,12]. In general, REEs are considered of average supply risk, low environmental implications and low-to-medium vulnerability to supply restrictions, and currently China is the leading producer and trader of REEs [3,13]. However, mining operators must now mitigate respective environmental impacts; thus, assessment of the toxicological hazard of the REEs for aquatic biota is needed [14]. Recently, it was reported that in REE-contaminated areas, phytotoxicity may be a concern [15]. It has been shown than lanthanum (La) accumulates in different organisms and thus may pose a hazard to species belonging to higher levels of the food chain [16]. In addition, people may receive elevated doses of REE via food [17,18]. Unfortunately, information on the environmental hazard for REEs other than cerium (Ce) and lanthanum (La) is limited and available data vary [1,16,19,20]. Even for La, very little information on toxicity to microorganisms has been published [16].

Concerning REOs, there is already a substantial amount of ecotoxicological data on CeO_2_ but not for other types of REOs [21,22]. Although CeO_2_ is used in a variety of applications, e.g., as a fuel additive [23], there is still no consensus on its hazard to living organisms [24].

Thus, analogously to REEs, information on toxicity of REOs to aquatic biota is very limited. It is important to note that REOs can be also produced as composites. For example, the physicochemical properties of metal oxides can be tuned via doping [25], i.e., introducing metals into the crystal structure of the oxides at the level of their synthesis often allows to widen the fields of applications of these oxides. The wide variety of composite oxides makes the evaluation of their case-by-case potential effects even more complicated and justifies the introduction of bioassays that allow for the evaluation of their toxic properties relatively cost-efficiently and rapidly.

Gonzalez et al. [1] in their recent review have emphasized the major challenges and research needs in ecotoxicology of lanthanides: (i) the chemical speciation of lanthanides in ecotoxicological test media must be taken into account as due to the formation of insoluble chemical species in majority of ecotoxicological test media, a remarkable underestimation of the lanthanides’ toxicity is a realistic scenario; (ii) mechanistic studies are needed to reveal possible existence of common mechanisms or modes of action across the lanthanide series.

The aim of the current paper was to address currently existing data gaps on ecotoxicity of REEs and REOs by providing toxicity data for five REEs (lanthanum, cerium, praseodymium, neodymium, and gadolinium) and nine (doped) REOs for two unicellular aquatic organisms (Gram-negative bacteria *Vibrio fischeri* and particle-feeding protozoa *Tetrahymena thermophila*). We also addressed the challenges/research needs pointed out by Gonzalez et al. [1] by (i) conducting the toxicity testing and experiments in media (deionized water for protozoa and 2% NaCl for *Vibrio fischeri*) that do not support formation of insoluble complexes (phosphates, carbonates and hydroxides of REEs), thus providing comparable toxicity data for different REEs and (doped) REOs; and (ii) studying whether the disturbance of biological membrane integrity is the main biological event in triggering toxic effects of REEs and REOs.

## 2. Materials and Methods

### 2.1. Synthesis and Characterization of Rare Earth Oxide (REO) Particles

The (doped) REOs studied in this work are listed and described in Table 1. The oxides were synthesized using spray pyrolysis technique [26]. The Brunauer–Emmett–Teller (BET) method was used for analysis of specific surface area (SSA) and for calculation of primary particle size of studied REO particles. Both techniques are described in detail in Joonas et al. [27].

### 2.2. Preparation and Characterization of REO Particle Suspensions

REO powder (~20 mg) was weighed and mixed with deionized (DI) water (18.2 MΩ, pH 5.6 ± 0.1, Milli-Q, Millipore, Billerica, MA, USA) to obtain a stock suspension of 1 g/L. The stock suspension was sonicated for 3 min (40 W, probe sonicator, Branson Ultrasonics Corporation, Danbury, CT, USA) and vortexed before use. Hydrodynamic size and ζ-potential of REO particles (100 mg/L) were measured in DI water using Malvern Zetasizer Nano-ZS (Malvern Instruments, Malvern, UK). The solubility of the only REO (La_2_NiO_4_), that proved toxic in our assays (see Section 3.3), was analyzed in two test media used in the toxicity assays of the current study: deionized water and 2% NaCl. For that, La_2_NiO_4_ stock suspension (1000 mg/L) was diluted with DI water or 2% NaCl to obtain concentration of 25 mg/L and let settle at room temperature for 24 h. La and Ni concentrations in the La_2_NiO_4_ were quantified with the total reflection X-ray fluorescence spectrometer (TRXF; Picofox S2, Bruker Nano GmbH, Berlin, Germany). For this, 1 mL of suspension was pipetted into 1.5 mL Eppendorf tube and the particles were pelleted by centrifugation at 20,000× *g* for 30 min. After centrifugation, 50 μL of the supernatant was carefully removed, mixed with gallium (Ga) internal standard (Fluka) in the ratio of 1:1 and 5 μL of this mixture was pipetted onto a quartz carrier disc. The concentration of metals was quantified with the Spectra software (version 7.2.5.0, Bruker Nano GmbH, Berlin, Germany).

### 2.3. Soluble Metal Salts Analyzed for Toxicity

The soluble metal salts tested in protozoan and bacterial toxicity tests were as follows: Ni(NO_3_)_2_·6H_2_O (Merck KGaA, Nottingham, UK, purity ≤ 100%), Co(NO_3_)_2_·6H_2_O (VWR, 98%), Gd(NO_3_)_3_·6H_2_O (Sigma Aldrich, Oslo, Norway, 99.9%), Sr(NO_3_)_2_ (Honeywell, Morristown, NJ, USA, 100%), Mn(NO_3_)_2_·6H_2_O (American Elements, Los Angeles, CA, USA, 100%), La(NO_3_)_3_·6H_2_O (Treibacher Industrie AG, Althofen, Austria, ≥95–100%), Ce(NO_3_)_3_·6H_2_O (Treibacher Industrie AG, ≥95–100%), Fe(NO_3_)_3_·9H_2_O (Sigma Aldrich, 99.99%), Pr(NO_3_)_3_·6H_2_O (Sigma Aldrich, 99.9%) Nd(NO_3_)_3_·6H_2_O (Sigma Aldrich, 99.9%). pH values of the stock solutions (1000 mg/L) were in the range of 4.5–5.6 (in DI water) and 5.2–6.6 (in 2% NaCl), except for Fe(NO_3_)_3_ that was more acidic (pH 2.1 in DI water and 2.3 in 2% NaCl).

### 2.4. Quantification of Reactive Oxygen Species

For further characterization, the abiotic reactive oxygen species (ROS) generation potential i.e., ability to generate ROS in DI water without the test organisms present, was determined for REO powders in the dark as described previously [28]. The REO particles were incubated at concentrations 1, 10 and 100 mg/L in triplicates on 96-well black polypropylene microplate for 24 h with two different dyes: (i) 2′,7′-dichlorofluorescein-diacetate (DCFH-DA, Life Technologies, Paisley, UK) that can detect wide variety of ROS, Mn_3_O_4_ NPs [28] (provided by University of Bremen, Bremen, Germany) were used as a positive control; and (ii) 3′-(p-hydroxyphenyl) fluorescein (HPF, Life Technologies) that is used to detect hydroxyl radicals, known •OH producing Fenton reaction (triggered by mixing 100 µmol/L FeSO_4_·7H_2_O (Reachim, analytical grade) with 1.47 mmol/L H_2_O_2_ (Sigma-Aldrich) was used as a positive control. 

### 2.5. Vibrio fischeri Kinetic Bioluminescence Inhibition Test (a Flash-Assay) in 2% NaCl and in PBS (Phosphate-Buffered Saline) Containing 2% NaCl

A kinetic acute bioluminescence inhibition assay (exposure time 30 min) with bacteria *Vibrio fischeri* was performed at room temperature (~20 °C) in 96-well microplates following the Flash-assay protocol [29]. Briefly, 100 μL of bacterial suspension was added to 100 μL of tested compound in the microplate well by automatic dispensing. Bacterial luminescence was continuously recorded during the first 30 s after dispensing (no additional mixing of the sample). After 30-min incubation, luminescence was recorded again. The Microplate Luminometer Orion II (Berthold Detection Systems, Pforzheim, Germany) controlled by the Simplicity version 4.2 Software was used. Reconstituted *V. fischeri* reagent (Aboatox, Turku, Finland) was used and all the chemicals and their two-fold dilutions were prepared in 2% NaCl. Each test was performed in 5–7 replicates. In each measurement series both negative (2% NaCl) and positive (3,5-dichlorophenol, 3,5-DCP) controls were included. Inhibition of bacterial luminescence (INH%) by the chemical was calculated as follows:

(1) $$\mathrm{INH}\text{\%}=100-\frac{{\mathrm{IT}}_{30}}{\mathrm{KF}\times {\mathrm{IT}}_{0}}\times 100$$

(2) $$\mathrm{KF}=\frac{{\mathrm{IC}}_{30}}{{\mathrm{IC}}_{0}}$$

KF (correction factor) denotes for the natural loss of luminescence of the control (i.e., bacterial suspension in 2% NaCl). IC_0_ and IT_0_ are the maximum values of luminescence during first 5 s after dispensing test bacteria to the control and test sample, respectively. IC_30_ and IT_30_ are the respective luminescence values after 30 min. EC_50_ is the nominal concentration of a compound reducing the bacterial bioluminescence by 50%. EC_50_ values were calculated from dose-effect data using REGTOX software (version EV7.0.5, Eric Vindimian, Paris, France) for Microsoft Excel^™^ (version 2010, Microsoft Corporation) [30].

For the evaluation of the speciation on bioavailability of REEs, we performed the *Vibrio fischeri* Flash assay in the medium containing phosphate, i.e., instead of 2% NaCl, PBS+NaCl was used throughout. Otherwise the assay was performed as described in the beginning of this Section. For that, PBS+NaCl medium was prepared: NaCl 19 g, KCl 0.2 g, Na_2_HPO_4_ 1.42 g, and KH_2_PO_4_ 0.24 g per litre DI water, pH = 7.4 (Cold Spring Harbor Protocols). Lyophilized bacteria were reconstituted as always in special diluent containing per litre: 20 g NaCl, 2.035 g MgCl_2_·6H_2_O and 0.3 g KCl. All toxicants were diluted in PBS+NaCl medium.

### 2.6. Vibrio fischeri Viability Assay (a ‘Spot Test’)

*Vibrio fischeri* viability assay (a ‘Spot test’) was performed as described in detail in Suppi et al. [31]. The assay evaluates the ability of the toxicant-exposed bacteria to form colonies on toxicant-free nutrient agar after 24-h exposure to the tested chemicals in 2% NaCl. Briefly, 100 μL of the *V. fischeri* suspension was added to 100 μL of varying concentrations of tested chemicals in 2% NaCl. Exponential (2-fold) dilution series of the REOs suspensions in the range of 1.5–500 mg compound/L and soluble metal salts in the range of 0.1–500 mg metal/L (depending on the toxicity of the compound; nominal concentrations) were tested. Incubation of bacteria with toxicants was performed in 96-well microplates (non-tissue culture treated, Greiner Bio-One) at 20–23 °C for 24 h without shaking, in the dark. After 24 h of exposure to the toxicants (or 2% NaCl), 3 μL of the cell suspension from each microplate well was pipetted as a ‘spot’ onto an agarized BH (Beneckea-Harvey) growth medium containing (per Litre): yeast extract 3 g, tryptone 5 g, glycerol (99%) 2 mL, NaCl 30 g Na_2_HPO_4_·12H_2_O 9.45 g, KH_2_PO_4_ 1 g, (NH_4_)_2_HPO_4_ 0.5 g, MgSO_4_·7H_2_O 0.3 g, agar 15 g). The inoculated agar plates were incubated for 72 h at 22–23 °C. Minimal bactericidal concentration (MBC) of the tested chemicals was determined as the lowest tested nominal concentration of a chemical which completely inhibited the ability of bacteria to form visible colonies after plating onto toxicant-free agar-plates. Each experiment was repeated at least three times.

### 2.7. Tetrahymena thermophila Viability Assay

Protozoan viability assay was conducted with *Tetrahymena thermophila* (strain BIII) using ATP concentration of the cell suspension for quantification of the viable biomass. For more details, see Jemec et al. [32]. Briefly, the cells were cultured in SSP medium supplemented with antibiotics penicillin G and streptomycin sulphate, and fungicide amphotericin B. The cells were harvested at logarithmic growth phase by centrifugation at 300× *g* for 5 min at 4 °C, washed in DI water and the culture density for viability assays was adjusted to 10^6^ cells/mL (OD_600 nm_ = 2). To assess the effect of REEs and (doped) REOs towards protozoa, 100 µL of *T. thermophila* cells in DI water were exposed to 100 µL of REE or (doped) REO formulations in DI water at different concentrations (final cell density 5 × 10^5^ cells/mL) on 96-well polystyrene plate for 24 h at 25 °C in the dark. Pure DI water with the same final density of *T. thermophila* culture served as a negative control. The pH of the samples varied in the range of 5–7 after 24-h exposure. All the concentrations were tested in at least triplicate; non-toxic (doped) REO particles (see Section 3.3.) were tested at least on two and REEs and dopants on at least three separate days. After the experiment, the cells were visualized with light microscope Olympus CX41 equipped with DP71 camera (Olympus Corporation, Tokyo, Japan). The viability was quantified by extracting ATP and measuring its content in the samples by applying luciferin-luciferase method described earlier [33].

### 2.8. Data Analysis

*V. fischeri* 30-min EC_50_ (the nominal concentration where bioluminescence inhibition was 50% compared to untreated control) and *T. thermophila* 24-h EC_50_ (the nominal concentration where based on ATP concentration the viability had decreased 50% compared to untreated control) with their respective 95% confidence intervals (95% CI) were calculated from the dose-response curves by applying the log-normal model of REGTOX software for Microsoft Excel^™^ [30]. For abiotic ROS generation, the differences between untreated and treated samples were assessed in R Statistical Software (version 3.2.3, R Foundation) using ANOVA followed by Tukey post-hoc test. All calculated results were considered statistically significant when 95% CI did not overlap or statistical test resulted in *p* < 0.05.

## 3. Results and Discussion

### 3.1. Physicochemical Characterization of Studied Rare Earth Oxide (REO) Particles

According to the primary size of the REO particles, four of the studied preparations could be considered nanoparticles (NPs) as their average particle size was <100 nm (Table 1). The hydrodynamic size and the ζ-potential of particles were characterized in two media: deionized water (DI) that was used as a test medium for protozoan viability assay and 2% NaCl that was a test medium for marine bacteria *Vibrio fischeri*. The hydrodynamic size of the REO particles in deionized water ranged from 147 to 285 nm (Table 1). As described in Joonas et al. [27], zeta potential of particles was measured after removal of large REO agglomerates by centrifugation, to obtain a reliable value and was ranging from −16.6 to +22.7 mV in DI water. It was not possible to measure hydrodynamic size and ζ-potential of particles in 2% NaCl as particles heavily agglomerated and settled. The pH values of the REO suspensions in DI and 2% NaCl were mostly neutral except for La_2_NiO_4_ and (La_0.6_Sr_0.4_)_0.95_CoO_3_ which were alkaline, pH from 8.6 to 9.5 (Table 1).

### 3.2. Analysis of Potential of REO Particles to Generate Reactive Oxygen Species (ROS) in Abiotic Conditions

**Figure 1:**
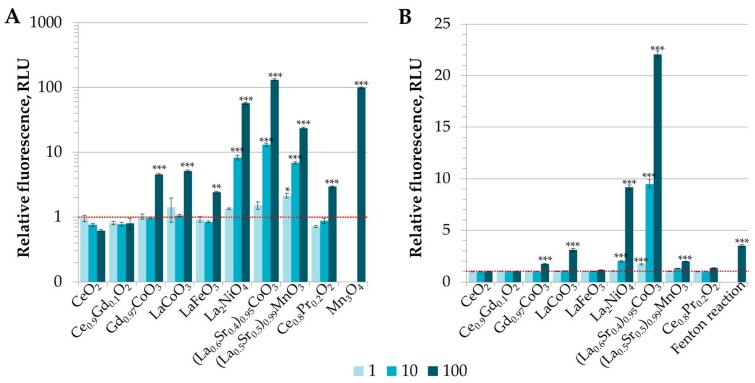
Abiotic generation of reactive oxygen species (ROS) by the rare earth oxide (REO) particles in deionized water measured after 24-h incubation with fluorescent dyes DCFH-DA (**A**) and HPF (**B**). Mn_3_O_4_ (**A**) and Fenton reaction (**B**) were included as positive controls. Concentrations are shown in the insets and are nominal, in mg compound/L. Dotted line indicates background fluorescence = 1.0. The asterisk (*) marks significant difference (*p* < 0.05); (**) highly significant difference (*p* < 0.01), and (***) very highly significant difference (*p* < 0.001) from the control.

Besides dissolution, another mechanism of action suggested for metal-containing particles, especially nanoparticles, is production of reactive oxygen species [21,34,35,36]. Indeed, most of the elements studied in this work (all lanthanides, Fe, Mn, Co, and Ni) are transition metals that due to their various oxidation states are considered to have oxidative capacity [36]. Thus, the ability of (doped) REO particles was studied in abiotic conditions, i.e., only in DI water with no test organisms present. After 24-h incubation, most of the doped REOs generated ROS as can be seen in Figure 1. The ROS production potency of REOs evaluated by the fluorescence of DCFH decreased in the order (La_0.6_Sr_0.4_)_0.95_CoO_3_ > La_2_NiO_4_ > (La_0.5_Sr_0.5_)_0.99_MnO_3_ > LaCoO_3_ > Gd_0.97_CoO_3_ > Ce_0.8_Pr_0.2_O_2_ > LaFeO_3_, while Ce_0.9_Gd_0.1_O_2_ and CeO_2_ did not increase the fluorescence of DCFH compared to the control (Figure 1A). Five doped REO particles could also produce •OH radicals and the order according to potency was similar: (La_0.6_Sr_0.4_)_0.95_CoO_3_ > La_2_NiO_4_ > LaCoO_3_ > (La_0.5_Sr_0.5_)_0.99_MnO_3_ > Gd_0.97_CoO_3_ (Figure 1B). These results indicate that dopants seem to have the dominant role in determining the oxidative potential of REO particles as all REOs containing cobalt, nickel or manganese as a dopant were capable of producing •OH radicals. In addition, the results were in accordance with study performed by Aruoja et al. [28] who showed that in abiotic conditions, Co_3_O_4_ and Mn_3_O_4_ in nanoform were capable of producing ROS (•OH radicals) even without photoactivation. Furthermore, although iron is a known triggerer of hydroxyl radical producing a Fenton reaction [37], LaFeO_3_ particles were least potent among ROS generating REOs and did not induce •OH radicals; similarly, Fe_3_O_4_ did not induce ROS in abiotic conditions in the study by Aruoja et al. [28]. Nickel is also a known producer of ROS in cells; however, the levels are usually lower compared to Fe and Co [38]. Although REEs did not seem to enhance ROS potency of REO particles in abiotic conditions at the same level as dopants, they still might have a role in organisms as Pagano et al. [39] showed that chlorides of some REEs like yttrium (Y), Ce and samarium (Sm) were capable of inducing excess ROS formation in sea urchin early embryos while others (La and Nd) produced ROS at similar levels to control. Similar to our results indicating that Ce-containing REO particles do not induce ROS (Figure 1), Ce NPs with a primary diameter of 3–5 nm and agglomerate size of 15–25 nm were shown to reduce ROS levels in A2780 ovarian cancer cells in vitro [40].

### 3.3. Toxicity Evaluation of REEs and (Doped) REOs

#### 3.3.1. Toxicity to Bacteria *Vibrio fischeri*

*Vibrio fischeri* are naturally luminescent Gram-negative marine bacteria that also are known under the name of *Photobacterium phosphoreum* NRRL-B-11177 and *Aliivibrio fischeri* [41]. *V. fischeri* rapidly responds to bioavailable toxicants with decrease in its natural bioluminescence in the time-scale of seconds-to-minutes, depending on the toxicant and its concentration, due to the disturbance of the integrity of cellular membrane, which functionality is essential for the central energy metabolism of the bacteria [42]. Therefore, the reduction of light output is a reflection of inhibition in bacterial metabolic activity and is proportional to the toxicity of the chemical or test sample [43]. The first bioluminescence inhibition assay using *V. fischeri* was commercialized already in 1979 as a Microtox™ (AZUR Environmental, Carlsbad, CA, USA) test and it is still probably the most widely used ecotoxicological test worldwide due to its low cost, rapidness, and great comparability as according to the literature, a lot of toxicity data for various chemicals have been obtained by applying this assay [41,44,45]. We have previously shown that as the suspensions of (nano) particles are often turbid due to insolubility and agglomeration of particles, a kinetic Flash Assay format of the *V. fischeri* bioluminescence inhibition assay [46] and not the format of conventional Microtox™ assay is better suited for toxicity evaluation of turbid suspensions of nanoparticles. The luminescence inhibition by toxicants is a sub-lethal response but it correlates well with the lethal endpoints for bacterium such as inability to grow on nutrient agar after exposure to the toxic concentration of the chemical [28]—a test format that was employed also in the current study for the evaluation of the minimal bactericidal concentration (MBC) of studied chemicals to *V. fischeri*. In addition, other test formats employing *V. fischeri* have been used for toxicity evaluation, such as inhibition of *V. fischeri* growth by toxicants in complex medium [47]. The average (from 2–5 experiments) toxicity values of REEs and (doped) REOs for *V. fischeri* obtained in this study are presented in Table 2. Altogether, data obtained with two different test formats are presented: 30-min luminescence inhibition assay (30-min EC_50_) and evaluation of the ability of bacteria to form colonies on agar media after 24-h incubation with toxicant (24-h MBC) (Table 2).

The EC_50_ values obtained from 30-min inhibition of luminescence toxicity test (Table 2) were slightly lower than previously reported for Ce and Gd analyzed in the *V. fischeri* assay (EC_50_ > 6.4 mg/L) [19]. This fact may be explained by different lanthanides compound tested (nitrates in the current study and chlorides in the work of González et al. [19]).

**Figure 2:**
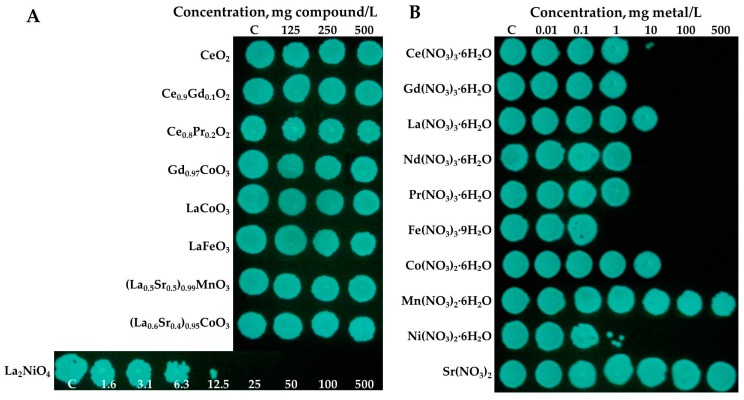
Bactericidal action of studied (doped) rare earth oxides (**A**) and salts of respective rare earth elements and dopants (**B**) to *Vibrio fischeri*. Bactericidal properties were evaluated by determining the colony-forming ability of the bacteria after exposure to suspensions of the particles in 2% NaCl for 24 h at room temperature, 22–23 °C. After exposure, 3 μL of bacterial suspension was transferred onto toxicant-free agarized nutrient medium and plates were incubated for 72 h at 22–23 °C. The concentrations are in mg compound/L (REO) or mg metal/L (REE salts), and nominal. Blue-green spots are bioluminescent bacterial colonies photographed in the dark. Minimal bactericidal concentration, MBC, is the lowest tested concentration that inhibited the bacterial ability to grow (form a colony on nutrient agar). MBC of La_2_NiO_4_ = 25 mg/L. For other REOs MBC > 500 mg/L. See also Table 2.

All the studied (doped) REOs were not toxic to *V. fischeri* in the bioluminescence inhibition assay even at the highest tested concentration, i.e., 30-min EC_50_ >500 mg/L (Table 2; Figure 2), showing that REOs are benign according to this test. Analogously, no toxic effects for CeO_2_ for *V. fischeri* was observed by Velzeboer et al. [48]: 15-min EC_50_ >100 mg/L. However, in the ‘Spot test’ after 24-h incubation La_2_NiO_4_ was bactericidal to *V. fischeri* already at 25 mg/L (Figure 2A; Table 2). Other REOs were not inhibitory in this assay (Figure 2A; Table 2). For the comparison, CuO and ZnO nanoparticles that are considered intrinsically biocidal [49] were toxic to *V. fischeri* in the same type of assays already in the concentration range of 1–10 mg/L [28].

**Figure 3:**
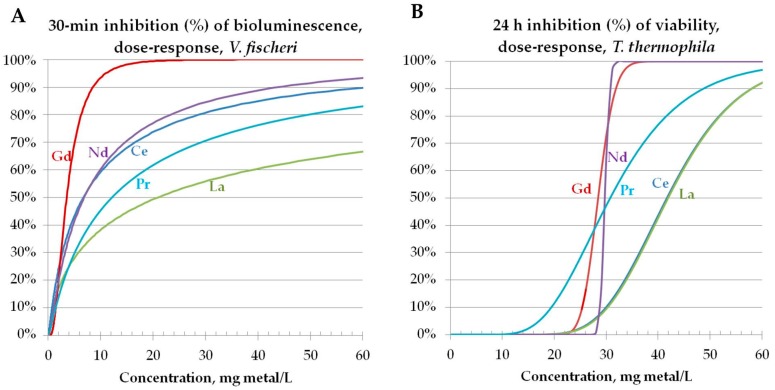
Toxicity of REEs to *Vibrio fischeri* (**A**) and *Tetrahymena thermophila* (**B**). Dose-response curves based on 30-min inhibition of bacterial luminescence (**A**) or cellular viability after 24-h exposure evaluated by ATP content of protozoan suspensions (**B**) are presented. The dose-response curves are constructed by combining the results from 2–5 different independent experiments (altogether 6–10 parallels). See also Table 2 and Figure 6.

Although REOs were benign to *Vibrio fischeri*, all studied REEs in their soluble form were toxic to bacteria (30-min EC_50_ 3.5–20.9 mg metal/L; 24-h MBC, 6.3–62.5 mg metal/L) (Figure 2B, Table 2). The respective dose-response curves are presented in Figure 3A. For the comparison, the toxicity of Cu and Zn ions to *V. fischeri* was 2.7 mg Zn/L and 0.42 Cu/L (30-min EC_50_ values; bioluminescence inhibition assay) [28].

**Figure 4:**
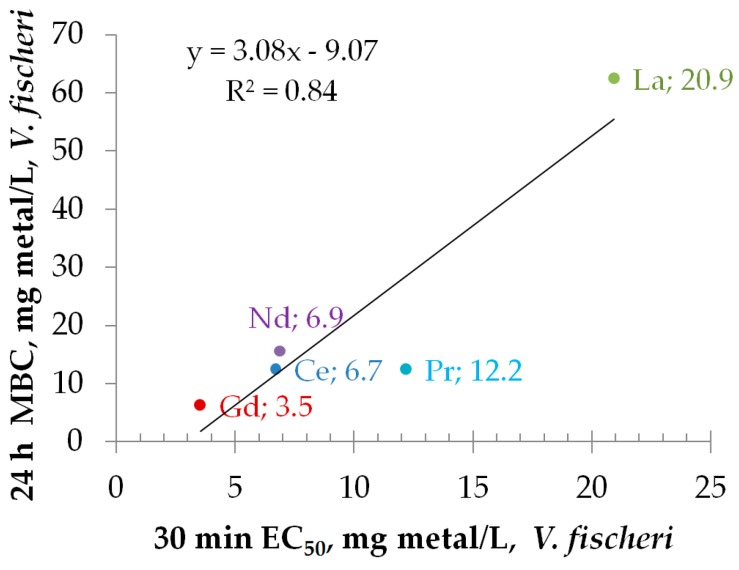
30-min EC_50_ versus 24-h MBC (*Vibrio fischeri*), mg metal/L. EC_50_ values were calculated from dose-response curves of 30-min inhibition of bioluminescence. 24-h minimal bactericidal concentration (MBC) is the lowest tested concentration to which bacteria were exposed for 24 h that prevented bacteria from growing (form colonies) after re-inoculation on nutrient agar. Tested metals are indicated as data labels accompanied by the respective 30-min EC_50_ values. See also Table 2.

In Figure 4, toxicity of soluble salts of Gd, Nd, Ce, Pr, and La in two different *V. fischeri* assay formats was compared. 30-min inhibition of luminescence is a sub-acute toxicity endpoint and evaluation of MBC is based on the toxic endpoint, i.e., the inability of the cells to grow following the exposure to toxic concentration of the chemical. Despite the endpoints are different, the 30-min EC_50_ and 24-h MBC values for lanthanides correlated reasonably well (R^2^ = 0.84) although the MBC values were on average 2-fold higher (Table 2; Figure 4).

In addition to REEs that are mostly the constituent metals of the studied REOs, also the soluble salts of the dopant metals (Co^2+^, Fe^3+^, Mn^2+^, Ni^2+^, Sr^2+^) were evaluated for their toxicity as these metals may be solubilized from the oxides and cause toxic effects [27]. In general, Co^2+^, Mn^2+^ and Sr^2+^ were not toxic (EC_50_ > 100 mg/L) or of relatively low toxicity in our assays; contrarily, Ni^2+^ was highly toxic in both tests where contact time with test organisms was 24 h (EC_50_ or MBC < 3 mg/L). Only Fe^3+^ proved very toxic in all three test formats (EC_50_ < 5 mg/L) but this effect cannot be attributed to acidic environment (although the stock solution was acidic; see Section 2.3) as in all tests the pH values at EC_50_ concentrations exceeded 5 (that did not affect the viability of selected microorganisms) (Table 2).

Our data on toxicity of iron and nickel are in accordance with the data from the literature. According to Storz and Imlay [50], the concentration of iron in the cells should be kept at low level as high concentrations of iron are toxic, mostly due to generation of reactive oxygen species (ROS). Sorokina et al. [51] have reported 30-min EC_50_ values for recombinant luminescent *E. coli* bacteria as 8.5 mg Fe^2+^/L and 1.3 mg Fe^3+^/L, i.e., quite similar to our data for *V. fischeri* (0.44 mg Fe^3+^/L) (Table 2). Concerning toxicity of nickel, its toxic effect to microorganisms is considered to occur (i) due to binding of Ni to catalytic residues of enzymes and also inhibiting enzymes allosterically; (ii) replacing the essential metals from metalloproteins; and (iii) causing indirectly oxidative stress although Ni is considerably weak generator of oxidative damage when compared to iron or copper [52].

#### 3.3.2. Toxicity to Protozoa *Tetrahymena thermophila*

*Tetrahymena thermophila* is a freshwater ciliated protozoan that, as a protist, is a predator of bacteria and prey for metazooplankton in aquatic food webs [53]. Like prokaryotic *V. fischeri*, *Tetrahymena* is also a unicellular model organism used in toxicology for decades [54]; but differently from *V. fischeri*, eukaryotic *T. thermophila* is capable of internalizing nano- and microscale particles by phagocytosis [55]. The latter ability makes it also suitable for the current study. Interestingly, *T. thermophila* is capable of surviving in DI water for at least as long as a week [56] due to its contractile-vacuole system, which maintains constant osmotic pressure in the cell [55]. This ability has been exploited in some recent studies [32,57] where *T. thermophila* was exposed to silver that forms easily insoluble chemical species in various test media as lanthanides do. Thus, to avoid lanthanide interactions with media components, the toxicity tests with *T. thermophila* were performed in DI water.

Although many of the studied REOs were capable of producing abiotic ROS (Section 3.2, Figure 1), analogously to *V. fischeri*, the REOs were not toxic to protozoa *T. thermophila* (24-h EC_50_ > 100 mg/L) (Table 2). The latter was not surprising as ROS-generating Ag nanoparticles proved to be toxic towards *T. thermophila* only by dissolving; this suggests that *T. thermophila* has efficient mechanisms to cope with oxidative stress [57]. For the comparison, CuO and ZnO nanoparticles were toxic to *T. thermophila* in the same type of assay in the concentration range of 1–10 mg/L [28]. Analogously to bacterial assays, all studied five REEs in their soluble form were toxic to protozoa (24-h EC_50_ from 28–42 mg/L) (Table 2; Figure 3B) being less toxic than Cu and Zn ions (24-h EC_50_ 7 mg Zn/L and 0.7 mg Cu/L [28]).

**Figure 5:**
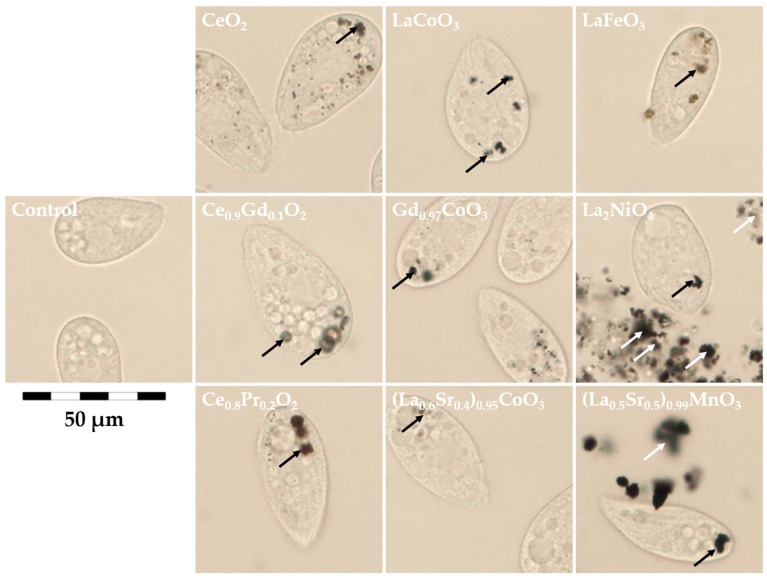
Viable protozoa *T. thermophila* exposed to 100 mg/L of various REOs for 24 h. Black arrows indicate food vacuoles filled with agglomerated REO particles, white arrows indicate particle-agglomerates released to the environment.

Despite *T. thermophila* was resistant to REO particles at concentrations up to 100 mg/L, visualization with microscope revealed that some of the food vacuoles were still filled with agglomerates of the REO particles after 24-h exposure (Figure 5). Previous studies with various particles have indicated that as soon as *T. thermophila* comes to contact with the particles, its food vacuoles start to fill up and after digestion, the pellets of agglomerated particles are excreted to the environment [28,33,58,59,60]. Alarmingly, compared to other freshwater invertebrates, *T. thermophila* has higher tolerance to heavy metals [21,49]; thus, although *T. thermophila* removes possibly toxic particles from the environment, the particles might still pose harm to more sensitive higher trophic levels that feed on protozoa during the REO particles are still inside the protozoan.

#### 3.3.3. Toxicity to Algae *Raphidocelis subcapitata* (Data Taken from the Literature): Comparison of Toxicity Pattern of REEs and REOs to Three Aquatic Species (Bacteria, Protozoa, Algae) 

The toxicity of the same set of REEs and REOs as in the current study was evaluated for the algae *Raphidocelis subcapitata* in the 72-h growth inhibition assay by Joonas et al. [27]. Differently from the toxic effects of REE to bacteria *V. fischeri* that somewhat varied (Table 2), the REEs were of comparable toxicity to algae (72-h EC_50_ values 1.2–1.4 mg metal/L) and were highly inhibitory to algal growth. The toxicity of REEs to protozoa was about 20–30 fold lower than to algae and toxicity to bacteria was about 10–20 fold lower than to algae, depending on bacterial toxicity endpoint. Out of the 5 REEs studied, Gd was the most toxic and La the least toxic lanthanide to bacteria *V. fischeri* and protozoa *T. thermophila*, supporting the proposed hypothesis that heavier lanthanides are more toxic than the lighter ones [61]. The very high toxicity of lanthanides toward algae (EC_50_ ~ 1 mg/L;) observed by Joonas et al. [27] was explained by authors by indirect effect of REEs via nutrient removal from the algal growth medium as a result of formation of insoluble REE’s phosphates. In the ‘Spot test’ in DI-water performed in parallel with the same algae, the 24-h MBC values of soluble salts of La, Ce, Pr, and Gd were much higher, 10 mg/L [27].

As described above, studied REOs were not toxic in protozoan viability assay and bacterial assays, except for La_2_NiO_4_ that was bactericidal to *V. fischeri* at 25 mg/L (Table 2; Figure 2A). Analogously, Joonas et al. [27] did not observe toxic effects for algae for the studied REOs in the ‘Spot test’ (MBC ≥ 100 mg/L) except La_2_NiO_4_ that was biocidal to algae at 10 mg/L and the authors proved that the toxic effect of La_2_NiO_4_ was due to dissolved Ni. Due to the toxicity of La_2_NiO_4_ in the 24-h growth inhibition assay for *V. fischeri* (24-h MBC = 25 mg/L; Table 2), we quantified the solubilized fraction of Ni and La in the suspension of 25 mg/L of La_2_NiO_4_. The concentration of soluble La in DI-incubated La_2_NiO_4_ was 0.081 mg/L and soluble Ni 0.56 mg/L. The respective concentrations in 2% NaCl incubated La_2_NiO_4_ were 0.087 mg La/L and 0.48 mg Ni/L. As the level of solubilized Ni (0.56 mg/L) was close to the 24-h MBC value (1.25 mg Ni/L), the solubilized Ni was assumingly the reason for the toxic effect of La_2_NiO_4_ (Table 2) as formerly proposed also by Joonas et al. [27].

One has to mention that in the algal growth inhibition assay also other REOs were adversely acting in lower concentrations compared to protozoan or bacterial assays (72-h EC_50_ values for algae were from 2.1 for (La_0.6_Sr_0.4_)_0.95_CoO_3_ to 98 mg/L for Ce_0.9_Gd_0.1_O_2_), but that effect was caused by entrapping algae into the agglomerates of the particles and restricting their growth [27].

Thus, it seems that the joint “opinion” of algae, protozoa and bacteria was that the REOs were not toxic to investigated aquatic species unless doped with toxic metals (such as Ni), the shedding of which may lead to toxic effects.

### 3.4. Effect of REEs on the Kinetics of Vibrio fischeri Bioluminescence

As shown in Table 2, all tested soluble REEs but not REOs proved toxic in the bioassays used in the current study. Most of the chemicals are toxic starting from a certain concentration due to the basal toxicity, i.e., affecting structures and functions common to all types of cells/organisms [62]. When we consider prokaryotic and eukaryotic cells (bacteria versus protozoa, for example), such a common structure is cell membrane. Moreover, in case of unicellular organisms the cell wall/membrane also separates the organism from the abiotic environment. As bacteria are unicellular organisms, they are protected from external, often hostile environment by strong cell wall [63]. Based on their cell envelope structure, the bacteria are classified into two broad groups: Gram-negative and Gram-positive. *Vibrio fischeri* is a Gram-negative bacterium. The biggest difference between Gram-negative and Gram-positive bacteria is concerning the peptidoglycan layer in the cell wall. The peptidoglycan of Gram-positive bacteria is 30 nm and in the Gram-negative bacteria just 2–3 nm thick but covered by another membrane—an outer membrane that is composed of phospholipids and lipopolysaccharides facing to the external environment [64].

As described above, the bioluminescence of bacteria *V. fischeri* is correlated with its metabolic activity and energetic metabolism—the process taking place inside the cell on cellular membrane. Indeed, the half-effective concentrations of chemicals that led to the inhibition of luminescence of *Photobacterium phosphoreum* (EC_50_) correlated reasonably well with octanol/water partition coefficients of the chemicals, acute L(E)C_50_ data from the ecotoxicological assays, in vitro toxicity data for animal cell lines and even with in vivo data for rats and mice [65].

**Figure 6:**
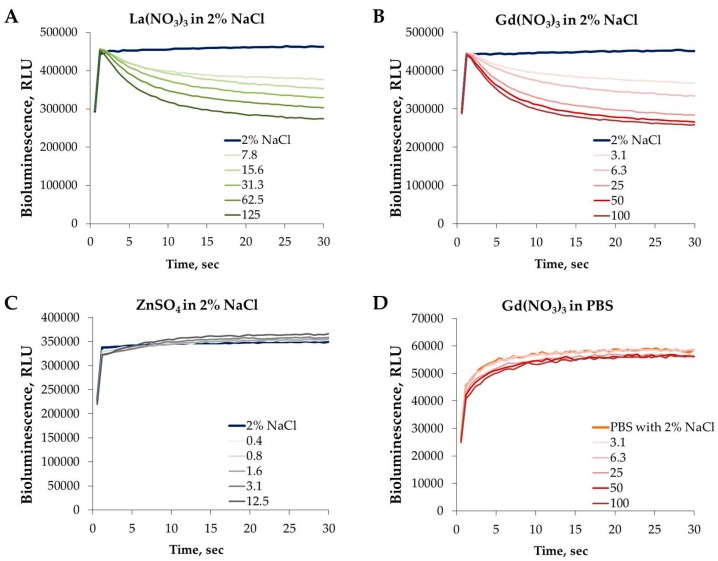
Kinetics of the *Vibrio fischeri* bioluminescence inhibition by La^3+^(**A**), Gd^3+^ (**B**,**D**), and Zn^2+^ (**C**) in 2% NaCl (**A**–**C**) and phosphate-buffered saline (PBS) containing 2% NaCl (**D**). Bacteria were exposed to different dilutions of soluble salts of Gd^3+^ and La^3+^ (rare earth elements), and Zn^2+^ and luminescence was followed during first 30 s of the contact with chemicals. Tested nominal concentrations (mg metal/L) are shown in the inset.

In addition, the time-scale and pattern of the kinetics of bacterial luminescence to the certain chemical allows comparison of the toxic action of different chemicals in respect of disturbance of the cellular membrane integrity [66]. As REEs have a very high affinity to phosphates, we hypothesized that the kinetics of the luminescence of *Vibrio fischeri* could be a very good mechanistic toxicity endpoint reflecting the early changes in the bacterial membrane (loss of integrity) due to exposure to REEs. The changes in bioluminescence of *V. fischeri* can be followed starting from the first seconds of the contact of bacteria with chemicals (Figure 6A,B). Although Figure 6 shows only the effects of Gd^3+^ and La^3+^, all five studied lanthanides were characterized by the rapidly acting (within 1st seconds of exposure; Figure 6A,B) inhibitory effect on bacterial bioluminescence (data not shown). For the comparison, exposure of bacteria to Zn (Figure 6C) did not induce analogous rapid changes in luminescence even at >10 mg Zn^2+^/L concentrations although the 30-min EC_50_ values for Zn and Gd are comparable (27 and 35 mg/L). The rapid decrease in bacterial bioluminescence upon exposure to REEs is probably due to the interactions between REEs and bacterial outer membrane. Indeed, according to Evans [61], the physiological properties of the lanthanides can be explained on the basis of their attachment to the outside of the cell membrane, with resulting disturbances in the cellular transport of metal ions. The strong attachment of positively charged lanthanide ions onto bacteria is also supported by negative ζ-potential of the *V. fischeri* cells (−21.8 mV; data not shown). Takahashi et al. [67] showed that phosphate and carboxylate groups are responsible for the adsorption of REEs on bacterial cell surface (Gram-negative *E. coli* and Gram-positive *Bacillus subtilis* were studied). The same conclusion was reached by Markai et al. [68] studying interaction of Eu with *B. subtilis* and by Texier et al. [69] for binding of Eu on Gram-negative bacteria *Pseudomonas aeruginosa*. Martinez et al. [70] in their studies with *B. subtilis* showed that light REE (La, Ce, Pr, Nd) had lower binding affinity than heavy REE (e.g., Tm, Yb, Lu). Ngwenya et al. [71] studied in more detail the lanthanide sorption sites on the Gram-negative bacterial surface using X-ray absorption spectroscopic measurements and EXAFS (Extended X-ray Absorption Fine Structure analysis) and suggested that the phosphate sites located on *N*-acetylglucosamine phosphate (a structural component of Lipid A in lipopolysaccharides, LPS, in the bacterial outer membrane) are assumingly the binding site of REEs. Interestingly, a positively charged deca-peptide antibiotic Colistin (polymyxin E) has somewhat similar mechanism of action by binding to the lipid A moiety and disrupting the integrity of the bacterial outer membrane resulting in cell death [72]. Importantly, Colistin is commercialized for the use in both human and veterinary medicine as the last line to combat infections caused by multidrug-resistant Gram-negative bacteria such as *Acinetobacter baumannii, Pseudomonas aeruginosa*, and *Klebsiella pneumoniae* [73].

### 3.5. Toxicity of REEs to Aquatic Organisms Depends on Speciation

One important factor affecting bioavailability of REEs is speciation: due to the formation of insoluble chemical species in majority of ecotoxicological test media the toxicity of lanthanides may be underestimated [1], and it is difficult to compare the toxicity data for different species obtained using different test media. On the other hand, when the speciation is reflecting the situation in the environment, the data are very relevant and informative for the environmental risk assessment.

We studied the effect of test media on the toxicity of REEs to *V. fischeri* by analyzing the effect of gadolinium on bioluminescence kinetics during 1st seconds of the exposure to gadolinium. As lanthanides have very high affinity to phosphates (they form insoluble phosphates) we tested the toxic effect of Gd nitrate in 2% NaCl (Figure 6B) and in phosphate buffered saline containing 2% NaCl (Figure 6D). It is evident that addition of phosphate converts gadolinium not toxic to *V. fischeri* by converting the ionic form of gadolinium to insoluble Gd phosphate.

Thus, although the soluble salts of lanthanides are all toxic, in the environmental settings they are rarely in the bioavailable (soluble) form as they form insoluble hydroxides, carbonates, phosphates, fluorides, and oxalates. Sulphates of lanthanides are sparingly soluble. For biology, the most important fact is that lanthanide phosphates and carbonates are insoluble under physiological conditions, i.e., pH 7.4 characteristic to body fluids [61]. However, at lower pHs the mobility of lanthanides (La, Gd, Ce were analyzed at pH 7.5, 5.5 and 3.5) in the soil increased [74]. Weltje et al. [75] studied the speciation and toxicity of trivalent lutetium (Lu^3+^) to *Vibrio fischeri* and showed that only free Lu^3+^ ions were toxic to *V. fischeri*. Also, within pH range 4.50–6.50 free Lu^3+^ was dominating species but in higher pH the free Lu^3+^ concentration rapidly decreased and at pH 7.5 free Lu^3+^ was reduced by about 80%.

Acidification in the vicinity of bacterial outer membrane where the excretion of the bacterial acidic metabolic by-products such as acetate [76] may locally acidify the environment is quite a realistic scenario. Phagocytosed REO particles may also exert toxicity due to modifications taking place in the acidic intracellular environment: lipid membrane dephosphorylation by shed metal ions from REO in acidic conditions of macrophage lysosomes has been suggested as the acute toxicity mechanism in TPH-1 cells (a human monocytic cell line) in vitro [77].

## 4. Conclusions and Outlook

Given the increased use of REEs and REOs worldwide, we studied the toxicity of a series of rare earth elements and oxides to marine bacteria *Vibrio fischeri* and freshwater protozoa *Tetrahymena thermophila*, to provide the necessary data for the ecotoxicological hazard evaluation of these compounds.

The results of the current study showed that lanthanide-based REOs did not pose a hazard to bacteria and protozoa, but use of toxic metals (such as Ni) as dopants in REOs may significantly decrease their environmental safety. We also showed that toxicity of REEs depends on speciation that leads to the variation of the toxicity values obtained from different assays performed in different test media.

Although soluble REEs were toxic to bacteria, we agree with Gonzalez et al. [19] who evaluated the toxicity of Ce, Gd, and Lu using a battery of aquatic species (algae, daphnids, rotifers, luminescent bacteria, hydra), that presence of lanthanides in the environment assumingly will not pose remarkable environmental risk except at some hotspots or for peak concentrations. We also provided an additional experimental proof on mechanism of toxic action of lanthanides (La^3+^, Ce^3+^, Pr^3+^, Nd^3+^, Gd^3+^) by disturbing the integrity of biological membrane.

We feel that the data obtained are important in the context of safety evaluation of REEs and REOs and are also informative for researchers working on e.g., new UV-visible wavelength semiconductor photocatalysts for pollutant removal in water as well as for evaluation of role of microbes in biogeochemical cycles.

## Figures and Tables

**Table 1:** Physicochemical properties of the (doped) rare earth oxide particles measured in 100 mg/L suspensions in deionized water (modified from Joonas et al. [27]).

Compound	Specific Surface Area (SSA), m^2^/g ^a^	Primary Size, nm (BET) ^a^	Hydro-Dynamic Size (nm) in DI (DLS) ^b^	ζ-Potential (mV) in DI ^b^	pH in DI ^c^	pH in 2% NaCl ^c^
Ce_0.9_Gd_0.1_O_2_	31.1	27	280	−16.6	6.3	6.3
LaFeO_3_	72	126	177	16.2	6.5	6.4
Gd_0.97_CoO_3_	34	230	166	18.8	6.3	6.3
LaCoO_3_	14	590	285	−17.5	6.7	6.4
(La_0.5_Sr_0.5_)_0.99_MnO_3_	7	137	194	−1.8	7.3	6.9
CeO_2_	22	38	172	8.5	6.5	6.5
Ce_0.8_Pr_0.2_O_2_	361	23	147	16.6	6.3	6.3
(La_0.6_Sr_0.4_)_0.95_CoO_3_	15	65	160	22.7	7.4	9.5
La_2_NiO_4_	3	284	266	−6.6	8.6	8.6

^a^ the Brunauer–Emmett–Teller (BET) method was used for analysis of the specific surface area (SSA) and for calculation of the primary particle size; ^b^ after centrifugation at 160× *g* for 10 min to remove large agglomerates DI- deionized water; ^a,b^ data taken from Joonas et al. [27]; ^c^ measured in the suspension of 1000 mg/L.

**Table 2:** Toxicity of (doped) rare earth oxide particles and soluble metal salts to protozoa *Tetrahymena thermophila* and bacteria *Vibrio fischeri*. The rare earth elements (REEs) in REOs are indicated in bold letters. The average EC_50_^a^ values with their 95% confidence intervals (in the brackets) are presented. *N* indicates the number of repetitive experiments performed to obtain the average value. For MBC ^b^ values, the representative value is presented (i.e., the value obtained in majority of experiments).

Compound	Toxicity Endpoint		
	24-h Decrease of Viability, %	30-min Inhibition of Luminescence, %	Ability to Yield Colonies on Agar Plates after 24-h Exposure to Chemical
	Protozoa *T. thermophila*	Bacteria *V. fischeri*	Bacteria *V. fischeri*
**(Doped) rare earth oxides**	EC_50_^a^, mg compound/L		MBC ^b^, mg compound/L
Ce_0.9_Gd_0.1_O_2_	>100; *N* = 2	>500; *N* = 2	>500; *N* = 2
LaFeO_3_	>100; *N* = 2	>500; *N* = 2	>500; *N* = 2
Gd_0.97_CoO_3_	>100; *N* = 2	>500; *N* = 2	>500; *N* = 2
LaCoO_3_	>100; *N* = 2	>500; *N* = 2	>500; *N* = 2
(La_0.5_Sr_0.5_)_0.99_MnO_3_	>100; *N* = 2	>500; *N* = 2	>500; *N* = 2
CeO_2_	>100; *N* = 2	>500; *N* = 2	>500; *N* = 2
Ce_0.8_Pr_0.2_O_2_	>100; *N* = 2	>500; *N* = 2	>500; *N* = 2
(La_0.6_Sr_0.4_)_0.95_CoO_3_	>100; *N* = 2	>500; *N* = 2	>500; *N* = 2
La_2_NiO_4_	>100; *N* = 2	>500; *N* = 2	25; *N* = 4
**Rare earth elements**	EC_50_, mg metal/L		MBC, mg metal/L
Ce(NO_3_)_3_·6H_2_O	41.7 (38.3–46.5); *N* = 3	6.70 (5. 81–8.48); *N* = 5	12.5; *N* = 3
Gd(NO_3_)_3_·6H_2_O	28.4 (27.3–30.6); *N* = 3	3.53 (3.38–3.76); *N* = 5	6.25; *N* = 3
La(NO_3_)_3_·6H_2_O	41.9 (38.0–47.2); *N* = 3	20.93 (17.07–25.94), *N* = 5	62.5; *N* = 4
Nd(NO_3_)_3_·6H_2_O	29.8 (29.5–31.2); *N* = 2	6.87 (6.66–8.57); *N* = 3	15.6; *N* = 4
Pr(NO_3_)_3_·6H_2_O	30.8 (26.6–35.8); *N* = 2	12.17 (11.08–14.82); *N* = 3	12.5; *N* = 4
**Dopant metals**	EC_50_, mg metal/L		MBC, mg metal/L
Fe(NO_3_)_3_·9H_2_O	4.9 (4.4–5.3); *N* = 3	0.44 (0.40–0.46); *N* = 6	1.25; *N* = 4
Co(NO_3_)_2_·6H_2_O	>100; *N* = 4	462 (404–564); *N* = 5	62.5; *N* = 5
Mn(NO_3_)_2_·6H_2_O	82.0 (76.9–90.4); *N* = 4	>500; *N* = 5	>500; *N* = 3
Ni(NO_3_)_2_·6H_2_O	2.7 (2.6–2.9); *N* = 3	>500; *N* = 5	1.25; *N* = 5
Sr(NO_3_)_2_	>100; *N* = 2	>500; *N* = 5	>500; *N* = 3

^a^ EC_50_—half-effective concentration; ^b^ MBC—minimal bactericidal concentration.
